# Supplementary material for: Seminoma and Embryonal Carcinoma Footprints Identified by Analysis of Integrated Genome-Wide Epigenetic and Expression Profiles of Germ Cell Cancer Cell Lines
Source: PLoS One. 2014 Jun 2;9(6):e98330. doi: 10.1371/journal.pone.0098330 (PMC4041891; doi:10.1371/journal.pone.0098330)
Supplement: File S1 — ZIP file containing DMRforPairs output for significant regions. Please start from the html files. (ZIP) [file pone.0098330.s008.zip › figures/10296.pdf]

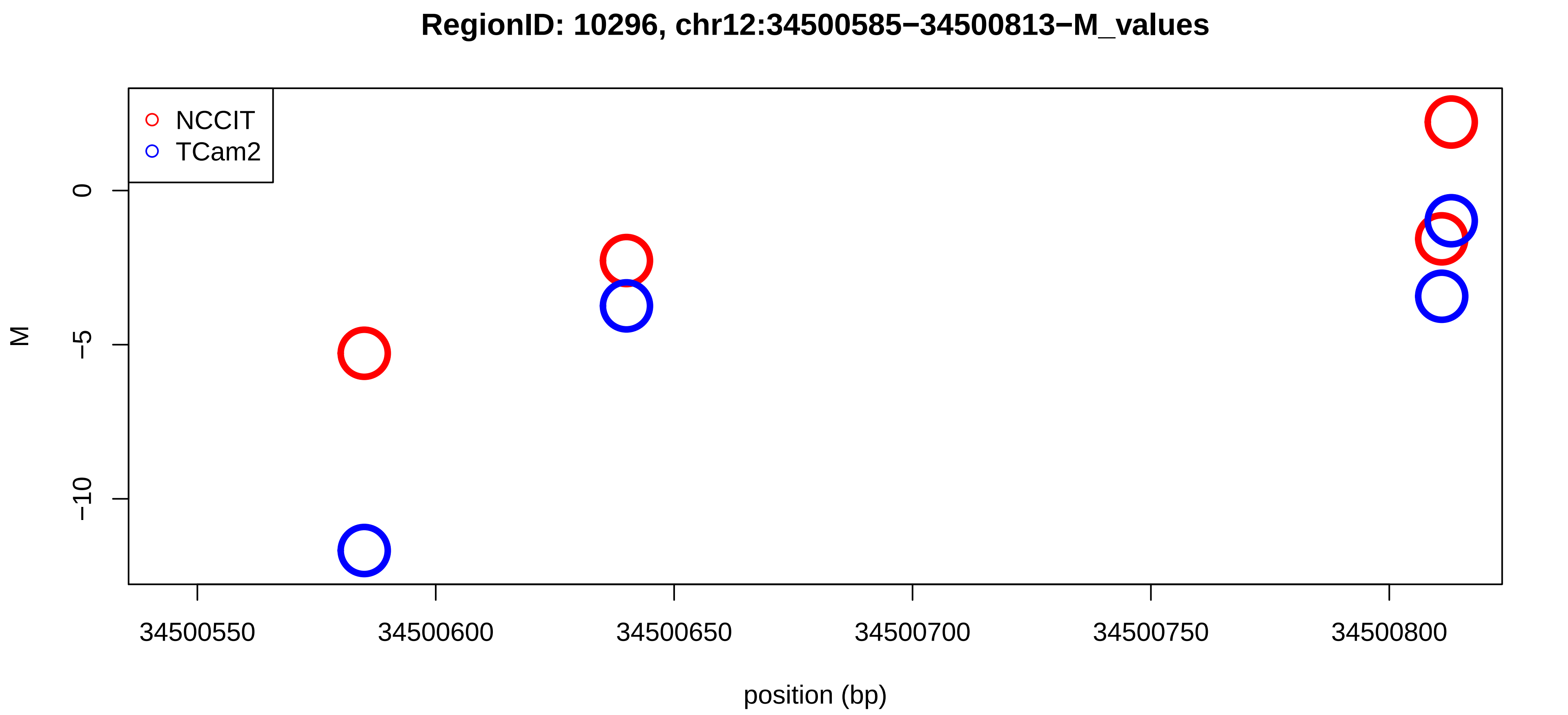

RegionID: 10296, chr12:34500585−34500813−M_values
NCCIT
TCam2
0
−5
−10
M
34500550
34500600
34500650
34500700
34500750
34500800
position (bp)

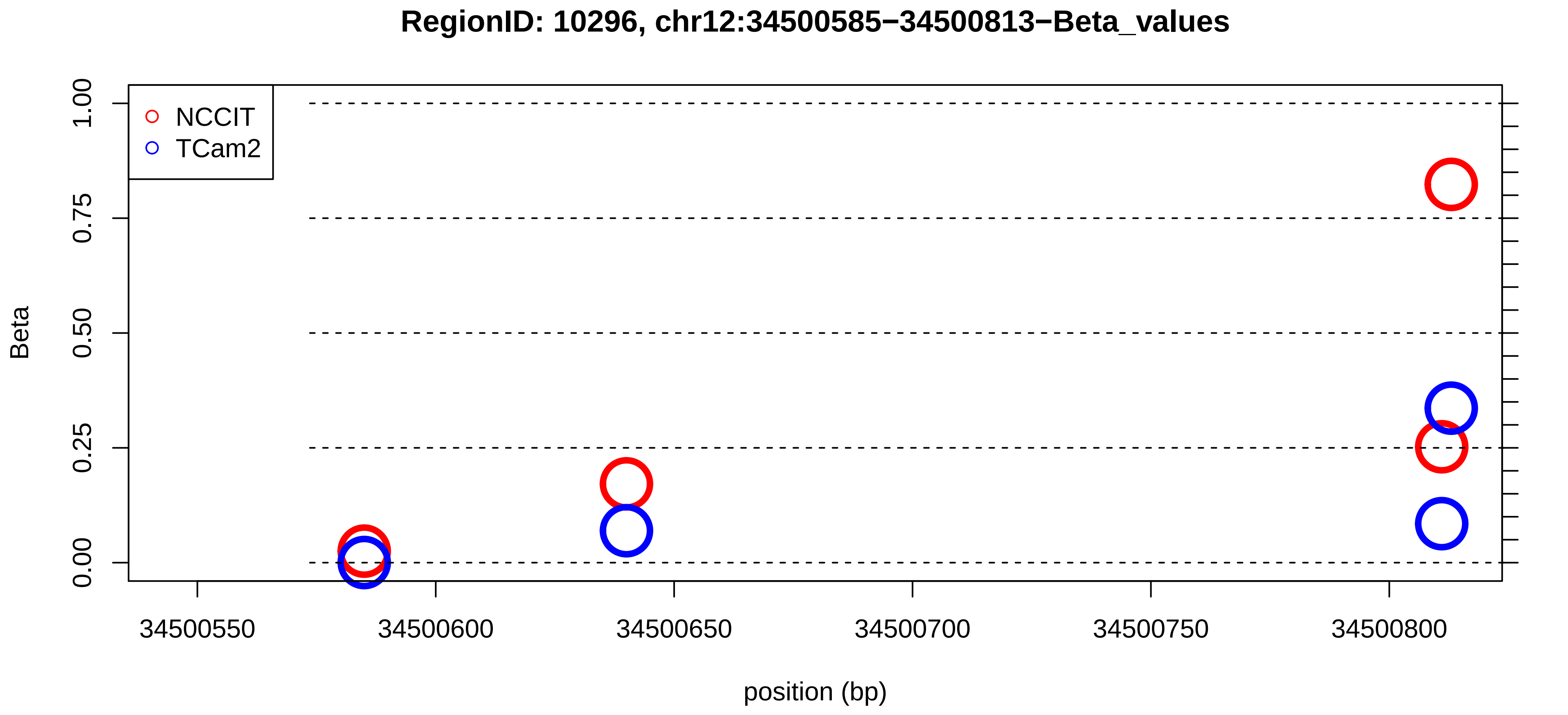

RegionID: 10296, chr12:34500585−34500813−Beta_values
NCCIT
TCam2
1.00
0.75
0.50
0.25
0.00
Beta
34500550
34500600
34500650
34500700
34500750
34500800
position (bp)
